# Immunocompetent Mice As a Model for Preclinical Studies of mRNA Vaccine Immunogenicity

**DOI:** 10.1134/S160767292370045X

**Published:** 2023-12-13

**Authors:** M. Yu. Shkurnikov, S. A. Tonevitskaya, E. V. Stepanova, S. A. Slobodov

**Affiliations:** 1 Shemyakin–Ovchinnikov Institute of Bioorganic Chemistry, Russian Academy of Sciences, Moscow, Russia; 2 Faculty of Biology and Biotechnology, HSE University, Moscow, Russia

**Keywords:** COVID-19, MHC-I, HLA, mRNA vaccines, S-protein

## Abstract

Conducting preclinical studies of mRNA vaccines is complicated by the lack of relevant animal models of the human immune system. Immunocompetent mice are widely used in biomedical research. However, critical differences in the genetics and immune system of mice and humans prevent the study of unique human immune responses in mice. Within the framework of this work, the possibility of modeling the cytotoxic T-cell response to mRNA vaccines encoding the S-protein of the SARS-CoV-2 virus was investigated. High-affinity peptides from S-protein were analyzed for the most frequent allelic variants of human MHC-I, two immunocompetent mouse lines (C57BL/6, BALB/c) and an outbred mouse model of IRC. The results of computer modeling have shown that mouse models can be used in preclinical studies of mRNA vaccines against SARS-CoV-2. Mouse MHC-I is able to present virus peptides that are highly affine for human MHC-I. Moreover, the immunogenicity of some of them has already been confirmed by examining blood samples from patients who have had COVID-19.

## INTRODUCTION

The COVID-19 pandemic has demonstrated the significant potential of a new type of vaccine, mRNA vaccines. The use of mRNA as a platform for vaccine development has several advantages over protein and peptide, killed and live attenuated vaccines, as well as DNA-based vaccines. First, safety: mRNA does not require integration into the genome, which reduces the potential risk of infection or insertional mutagenesis to zero. In addition, the mRNA vaccine is processed inside the cell by standard cellular enzymes along with endogenous mRNA, and its half-life in vivo can be controlled using various modifications and delivery methods [1]. Immunogenicity inherent of mRNA can be reduced to further improve the safety profile [2]. Second, efficiency: various modifications make mRNA more stable and easily translated [2]. Efficient delivery in vivo can be achieved by incorporating mRNA into carrier molecules that ensure rapid uptake and expression in the cytoplasm [3]. Third, the scalability of production: mRNA vaccines allow easy increase production due to high yields of in vitro transcription reactions.

A characteristic feature of antiviral immunity created by mRNA vaccines is the formation of a cytotoxic T-cell response. Effector CD8+ T lymphocytes play the key role in antiviral immunity at the initial stages of COVID-19 [4]. Major histocompatibility complex (MHC) class 1 molecules determine the efficiency of presentation of COVID-19 antigens. Immediately after entering the cell, SARS-CoV-2 induces the translation of its proteins. Some of them enter the proteasomes of the infected cell; they are cleaved to peptides 8–12 amino acid residues long and bind to MHC class I molecules. After binding, the complex consisting of the MHC class 1 molecule and the viral peptide is transferred from the Golgi complex to the cell surface, where it can be recognized by the CD8+ T-cell receptor of the effector T-lymphocyte. In response to the interaction, the CD8+ T-lymphocyte is activated and destroys the infected cell using perforins and serine proteases [5].

Preclinical studies of mRNA vaccines are complicated by the lack of relevant animal models of the human immune system. Immunocompetent mice are widely used in biomedical research. However, critical differences in the genetics and immune systems of mice and humans preclude the study of unique human immune responses in mice. For example, in humans, MHC class 1 molecules are encoded by the *HLA-A*, *HLA-B*, and *HLA-C* genes, each of which can be represented in two variants (alleles). In the human population, there are hundreds of variants of each allele encoding class 1 MHC molecules with an individual ability to interact with foreign peptides. In mice, MHC class 1 molecules are encoded in the H2 region, which includes three genes: *H2-K*, *H2-D*, and *H2-L*. However, the number of allelic variants of these genes is quite scarce, and the majority of mouse model lines are homozygous, which greatly reduces the repertoire of MHC class 1 molecules [6].

In the framework of this work, we studied the possibility of modeling the cytotoxic T-cell response to mRNA vaccines encoding the S-protein of the SARS-CoV-2 virus. High-affinity peptides from the S-protein were analyzed for the most common human MHC class 1 allelic variants, two immunocompetent mouse lines (C57BL/6 and BALB/c), and an outbred IRC mouse model.

## MATERIALS AND METHODS

The BALB/c mouse line is homozygous for MHC class 1 alleles and has the H-2Kd, H-2Dd, H-2Ld genotype. The C57BL/6 line is homozygous for the H-2Kb and H-2Db alleles. Outbred IRC mice carry much more MHC class 1 alleles: H-2Db, H-2Dd, H-2Dq, H-2Kb, H-2Kd, H-2-Kk, H-2Kq, H-2Ld, and H-2-Lq. When evaluating the high-affinity peptides of the SARS-CoV-2 S protein for the human population, we analyzed the most common MHC class 1 allelic variants in the Moscow region: HLA-A*02: 01, HLA-C*12: 03, HLA-A*03: 01 , HLA-A*01: 01, HLA-A*24: 02, HLA-B*07: 02, HLA-B*08: 01, HLA-B*18: 01, HLA-C*07: 02, HLA -C*06: 02, HLA-C*07: 01, and HLA-C*04: 01 [7].

To assess the affinity of interaction with MHC class 1 molecules, we used the S-protein sequence of SARS-CoV-2 variant Wuhan-Hu-1, which was published on the GISAID portal [8]. For each amino acid of the S protein, the probability of cutting by the proteasome at a given position was calculated using the NetChop software [9]. The list of viral peptides was defined as a set of various S-protein fragments consisting of 8–12 amino acids with a proteasome cleavage probability of at least 0.1 from each end of the fragment.

The binding affinity of viral peptides to MHC class 1 molecules encoded by all of the above alleles was assessed using the netMHCpan software [10]. Peptides with a low binding affinity for all alleles from the given population were excluded. The threshold value characterizing low affinity was 500 nM.

The proportion of peptides from the RBD domain of the SARS-CoV-2 S protein was compared using Fisher’s exact test. Data processing and statistical analysis were performed in the R software environment.

## RESULTS AND DISCUSSION

**Fig. 1.**
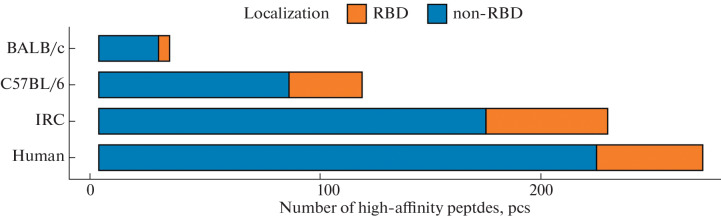
Number and localization of the origin of SARS-CoV-2 S-protein peptides with high affinity for different populations.

Taking into account the fact that the composition of the catalytic subunits of the 20S particle of the proteasome is highly homologous between mice and humans [11], the NetChop neural network version 3.1 was used for computer simulation of the proteasomal degradation of the SARS-CoV-2 S protein. Modeling showed the possibility of forming 273 peptides with an affinity of interaction with one of the variants of the MHC class 1 molecule less than 500 nM. Of these, 48 peptides were derived from the most variable part of the S protein, the RBD domain (Fig. 1). C57BL/6 mice are able to present 119 peptides (33 from the RBD domain) on MHC class 1 molecules, and BALB/c mice are able to present only 32 peptides (5 from the RBD domain) on MHC class 1 molecules. The largest number of viral peptides—230 peptides (55 from the RBD domain)—can be presented by IRC mice. Previously, it was shown that the number of predicted high-affinity peptides correlates with the objective number of T-cell responses of CD4^+^ and CD8^+^ T lymphocytes [12, 13]. It can be concluded that mouse MHC class 1 molecules can present a significant number of SARS-CoV-2 S-protein peptides with a high affinity for humans.

It should be noted that the proportion of high-affinity peptides from the RBD domain in the C57BL/6 mice was significantly higher than in humans (odds ratio: 1.8, *p* = 0.03). In turn, the proportion of high-affinity peptides from the RBD domain in outbred IRC mice and BALB/c mice corresponded to the value characteristic for humans.

**Fig. 2.**
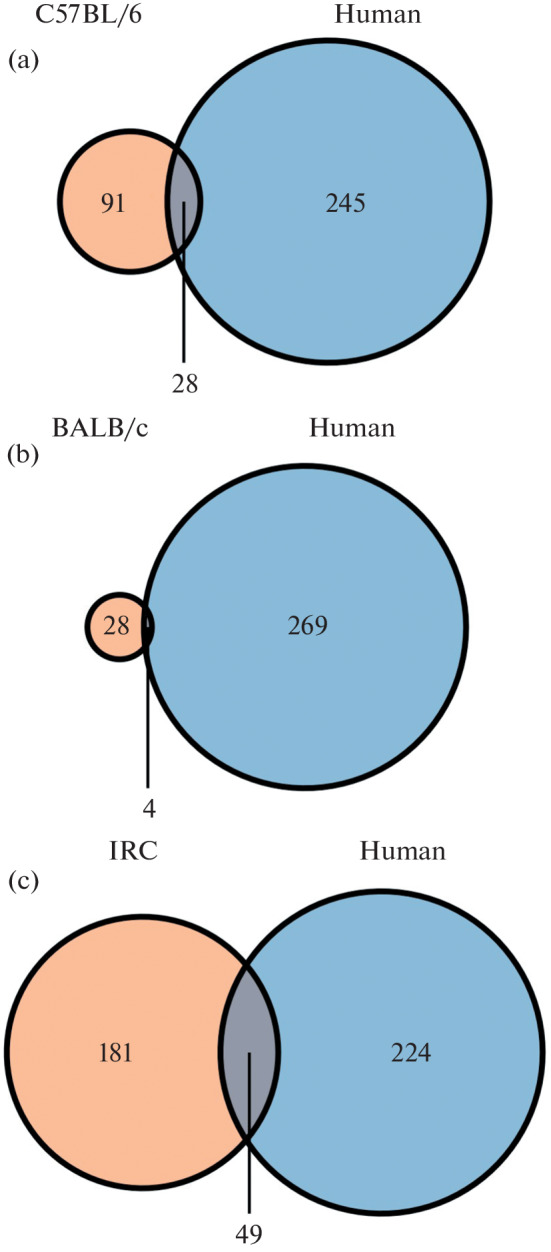
Number of high-affinity peptides presented by MHC class 1 of both mice and humans: (a) comparison of C57BL/6 mice and humans; (b) comparison of BALB/c mice and humans; (c) comparison of IRC mice and humans.

The proportion of viral peptides that can be presented by MHC class 1 molecules in both mice and humans was also estimated (Fig. 2). It ranged from 1.3% in BALB/c mice to 10.8% in IRC mice. Despite the low proportion of such peptides relative to humans, it should be noted that they account for 12 to 24% of S-protein peptides that can be presented by MHC class 1 molecules of the corresponding mouse line.

The analysis of the IEDB database showed that the C57BL/6 and IRC mice can present three immunodominant epitopes: VGYLQPRTF [14], VTWFHAIHV [15], and SIIAYTMSL [16]. These epitopes do not belong to the RBD domain of the SARS-CoV-2 S protein. According to the T-cell COVID-19 Atlas portal, VGYLQPRTF and SIIAYTMSL epitopes are conserved in actual virus strains [17]. However, the VTWFHAIHV epitope is absent in the Omicron BA.1 and BA.3 variants due to the A67V mutation and the HV 69–70 deletion.

The results of computer simulation showed that mouse models can be used to assess the CD8^+^ cytotoxic response in preclinical studies of mRNA vaccines against SARS-CoV-2. Mouse MHC class 1 can present viral peptides that exhibit high affinity for human MHC class 1. Moreover, the immunogenicity of some of them has already been confirmed in the study of blood samples from patients who recovered from COVID-19 [14–16]. It should be noted that the K18-hACE2 mouse model, which is widely used in the studies of SARS-CoV-2, was derived from the C57BL/6 line and carries the H-2Kb, H-2Db genotype. The features of the presentation of the virus peptides by the major histocompatibility complex of this mouse line should be taken into account when analyzing the results of experiments.
